# A Virtual Reality-Based Automated Perimeter, Device, and Pilot Study

**DOI:** 10.1167/tvst.10.3.20

**Published:** 2021-03-22

**Authors:** Mario Montelongo, Alberto Gonzalez, Freddy Morgenstern, Sean P. Donahue, Sylvia L. Groth

**Affiliations:** 1 WESMD PA, San Antonio, TX, USA; 2 Olleyes, Inc., Summit NJ, USA; 3 Department of Ophthalmology and Visual Sciences, Vanderbilt University, and Vanderbilt Eye Institute, Vanderbilt University Medical Center, Nashville, TN, USA

**Keywords:** automated perimetry, virtual reality, head mounted device, visual field

## Abstract

**Purpose:**

The purpose of this study was to describe a novel, virtual reality (VR)-based platform for evaluating visual fields.

**Methods:**

Three subjects were tested on the VisuALL VR headset. Data collected included test duration per eye, total fixation losses (FLs), total false positives (FPs), and total false negatives (FNs). Mean threshold values were collected from the superior temporal (ST), superior nasal (SN), inferior nasal (IN), inferior temporal (IT) quadrants, and from the central 12 degrees (central), 12 to 24 degrees (pericentral), and from all testing loci (global).

**Results:**

Six eyes of 3 subjects (2 men, 1 woman; mean age 30 years) were tested using the T-24 protocol (a Humphrey visual field [HVF] 24-2 equivalent). Mean test duration was 4.43 ± (SD) 0.11 minutes/eye. Mean threshold values ± SD for ST, SN, IN, IT, global, central, and pericentral were 31.1 ± 0.95 decibel (dB), 31.9 ± 0.3 dB, 32.0 ± 0.3 dB, 32.0 ± 1.1 dB, 31.9 ± 0.5 dB, 32.8 ± 0.5 dB, and 31.5 ± 0.5 dB, respectively.

**Conclusions:**

This work describes the technical aspects of the VisuALL. Participants were able to complete the test and generate threshold values at each of 50 locations in the central 24 degrees of visual field. This VR-based visual field test shows potential to become an alternative to analog, stationary standard automated perimetry tests.

**Translational Relevance:**

The VisuALL is an immersive, VR-based, automated perimeter that effectively addresses some of the limitations inherent to other popular perimetric devices. Potential advantages of the VisuALL are its adaptability, portability, and efficiency for patients. This device may be able to fill the gap present in at-home glaucoma monitoring and expand the reach of glaucoma management.

## Introduction

In clinical ophthalmology, visual field (VF) testing provides invaluable information regarding the integrity of the afferent visual pathways. VFs can be used to localize central nervous system abnormalities, such as brain tumors, strokes, and infiltrating disease, and aid in evaluating changes of these entities. Their use is mandatory for managing common eye diseases, such as glaucoma and other optic neuropathies. Standard automated perimetry (SAP) is the most common form of VF testing, with the most popular devices on the market being the Humphrey Field Analyzer (HFA; Zeiss Meditech, Dublin, CA) and the Octopus (Haag Streit International, Koeniz, Switzerland). SAP determines the visual threshold for light detection of static stimulus at various locations throughout the retina. The higher the attenuation of the threshold value at each testing point, the more sensitive the retina. This contrasts with kinetic perimetry, which determines retinal sensitivity by utilizing moving stimulus of varying intensity and size across a background. We will be focusing our discussion on SAP.

The most frequently used testing strategies (Swedish Interactive Thresholding Algorithm [SITA] Standard and the Tendency Oriented Perimetry [TOP]) measure light detection most commonly at locations 24 to 30 degrees from fixation. SITA Standard systematically identifies the threshold values of four “anchor” points by a double crossover method, involving crossing the threshold value twice to determine the sensitivity for that point. It then uses maximum posterior probability calculations in adaptive VF models to estimate threshold values for adjacent points.^1^^,^^2^ Test time is longer when patients give inconsistent responses, have slower response times, or when field defects are present. The TOP strategy is based on testing each position only once but interpolating the information to the surrounding areas.^3^

The HFA's ubiquity yields largely interchangeable VF reports between provider offices. This is particularly helpful in assessing glaucoma due to its slowly progressive nature. There are well-validated glaucoma progression analysis algorithms in the HFA,^4^^,^^5^ which assist in detecting progression over time. Repeat testing on a single device is beneficial due to a known learning effect.^6^^,^^7^

Although the HFA and Octopus are the most widely used instruments, they also have inherent limitations. The machines are large and immobile. This confinement to a single location restricts their use to patients healthy enough to present to their clinic. Additionally, test reproducibility is highly dependent on a patient's head position – a problem addressed by providing a forehead and chin rest. However, patients with reduced cervical flexibility due to arthritis, neck fusion surgery, kyphosis, or other range-limiting conditions often find the positioning challenging, if not impossible. Inadequate positioning during SAP can lead to lens rim artifacts and accentuate the defect caused by ptosis. A patient's discomfort may negatively influence the HFA's reliability and inflate fixation errors, and the percentage of false positive, or false negative responses. Long test duration can challenge patients’ endurance and concentration; this is particularly problematic for the elderly who are most likely to need these tests. Furthermore, patients’ negative feelings toward VF testing are well-documented. A recent study found that among the commonly performed diagnostic tests in an ophthalmology office, VF protocols ranked as the worst in popularity.^8^

When considering an ideal platform for VF testing, adaptability, comfort, and engagement are virtues. These characteristics can be seen in the audio-visual-spatial technology of extended reality (XR). The term XR is an umbrella term, covering three types of imaging: virtual reality (VR), augmented reality (AR), and mixed reality (MR). All three types of XR create a new environment with which the user can interact but differ in the source of the images used. VR creates completely new virtual images and is gaining popularity in games and entertainment, whereas AR adds graphics to live images of the real world (filters used in Snapchat [Snap Inc., Santa Monica, CA]). MR combines real and simulated images.

These concepts may seem remote from eye care but can in fact provide an interactive environment to facilitate VF testing. Improved patient comfort and engagement provided by VR may lead to more reliable results and higher patient satisfaction. This paper describes a novel VR-based visual function platform.

## Materials and Methods

### Device

The VisuALL (Olleyes, Inc., Summit, NJ) is a novel, US Food and Drug Administration (FDA)-registered, VR-based, VF platform. It was designed to emulate the other commonly used automated perimeters and is composed of two parts: the hardware and software.

**Figure 1.**
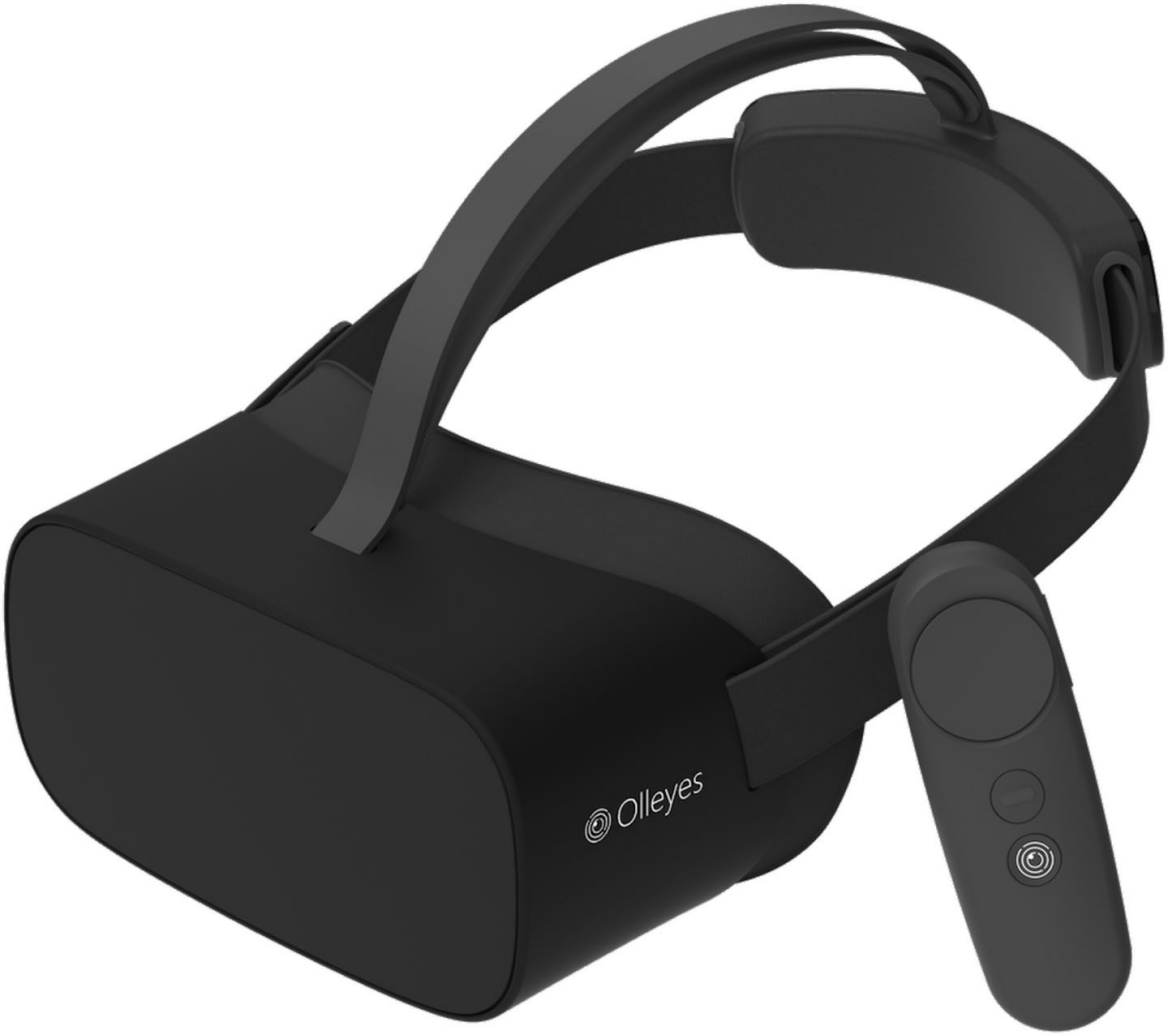
The VisuALL head mounted device (HMD) and Bluetooth connected handpiece.

**Figure 2.**
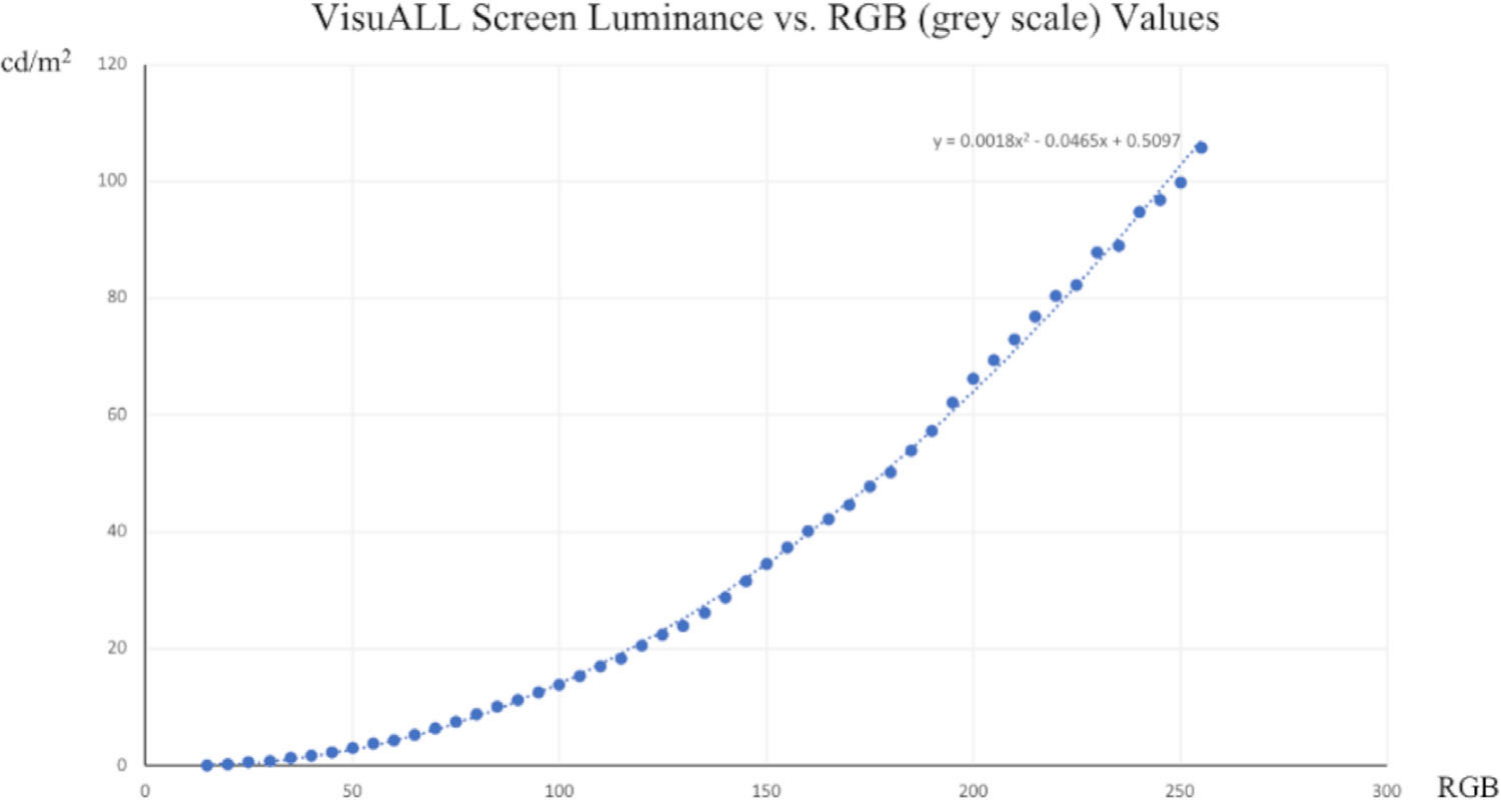
VisuALL screen luminance (cd/m^2^) versus RBG (gray scale) values.

The hardware includes three main components: a head mounted device (HMD) also known as a VR headset (Fig. 1), a web-capable device (laptop, phone, or tablet) and a Bluetooth connected handpiece (see Fig. 1). The HMD is powered by Pico (Pico Interactive, Inc., San Francisco, CA). It weighs 276 g and includes a Quad High Definition Liquid Crystal Display with a resolution of 3840 × 2160 pixels and a refresh rate of 75 Hz. The display is divided into two halves (one for each eye) with a resultant resolution of 1920 × 2160 pixels on each half. The display measures 125.4 × 70.56 mm and is placed at a distance that subtends a field of view (FOV) up to 100 degrees. A polyurethane insert is used to create a barrier to prevent ambient light from entering the field of vision as well as to provide comfort and support for the user (Fig. 2). The entirety of the HMD can be sanitized using alcohol preparation pads after each use.

The HMD includes several tracking systems, inertial measurement units (IMUs) consisting of gyroscopes and accelerometers, and infrared-based (IR) position tracking with two arrays of six IR sensors.

To measure the luminance, the HMD was fixed in a horizontal position and a Mavomonitor USB Luminance Meter (Hotek Technologies, Inc., Yelm, WA) was superimposed on the right HMD lens. The luminance values were obtained from a circumference with a diameter of 500 pixels located in the center of the tested screen. The luminance test was performed in a room with all lighting switched off.

The VisuALL software uses a gray scale (red, green, blue [RGB] scale) for display adjustment. Forty-nine central circumferences were shown with variable pixel intensities (5-pixel intensity interval) between 0 pixels (black, 0 cd/m^2^) and 255 pixels (white, 120 cd/m^2^, 0 db). The values between each measurement were linearly interpolated (see Fig. 2)

The VisuAll software includes the Olleyes cloud-based server, the VisuALL web application, and the Unity algorithms. The cloud, unity, and web applications are all Health Insurance Portability and Accountability Act (HIPAA) compliant.

### Unity Application

The HMD runs the VisuALL Patient Testing Application (PTA), which was written in Unity (a cross-platform gaming engine; Unity Technologies, San Francisco, CA). By leveraging this engine, VisuALL is able to create a fully immersive and self-contained environment to perform self-directed, interactive training tutorials for the patient and then proceed to the VF test.

Another key component of the VisuALL PTA is the proprietary thresholding algorithms, which implement complex decision trees to determine most efficiently what the patient's threshold for light detection is at each location.

For increased security and for HIPAA compliance reasons, the patient data is not stored in the HMD and is instead stored in a cloud-hosted backend.

### Web Application

The HMD can be operated with the VisuALL WebApp using any web-capable device. Once in the WebApp, the test administrator can input a patient's personal information, enter testing parameters, and select the specific VisuALL HMD to be used. If a test has been previously completed, reports can be reviewed through the WebApp. An internet connection is required to start a test. Once a test has commenced, a connection is no longer needed.

### Backend APIs

Both the VisuALL PTA and the WebApp leverage the backend APIs to store and retrieve patient and test data securely as well as for reporting and test statistics calculations. All the VisuALL data is stored in HIPAA-compliant cloud services hosted at Microsoft's Azure (Microsoft Corp., Redmond, WA) data centers. This permits the system to leverage multiple cutting-edge technologies (Machine Learning, multi-region Kubernetes clusters, ephemeral/on-demand Serverless Compute, etc.) in a secure and cost-effective manner.

The APIs also provide mechanisms to process the patient test data and generate reports with the test results for the healthcare professionals to interpret. The reports include the traditional plots/charts along with the standard statistical calculations that are commonly used by healthcare professionals (median deviation [MD], pattern standard deviation [PSD], total deviation, pattern deviation, etc.).

### Testing Protocols

The VisuALL uses Goldmann size III test stimuli in each VF protocol and tests both eyes simultaneously. The device uses scotopic 1 cd/m^2^ testing conditions in which a white stimulus is shown against a black background (1 cd/m^2^). It uses a double crossover method to establish the threshold for four anchor points (one in each quadrant) after which it uses proprietary testing strategy to determine the threshold values for predetermined adjacent locations. The T-24 protocol tests 50 points of the central 24 degrees (with test locations 6 degrees apart) and the T-10 tests 68 points of the central 10 degrees (with test locations 2 degrees apart). Threshold values are reported in decibels (dB) in a range of 0-49. Fixation losses are determined by using the Heijl-Krakau strategy, which involves mapping the physiologic blind spot at the beginning of the test and periodically testing this spot as the test progresses. If a response is elicited by a stimulus in this location, it is recorded as a fixation loss.^9^ False negatives are measured by showing the user a stimulus that is 0.5 cd/m^2^ lower intensity than previously seen; a false negative is recorded if the hand piece is not clicked. There is also a suprathreshold version of the T-24, the S-24. The suprathreshold test is the only protocol that tests each location only once, using a stimulus of constant, predetermined intensity and uses response time as an outcome measure.

After testing is complete, the software generates a report that includes the patient's name, date of birth, gender, test ID, examination date, test time, and test strategy. It also lists the fixation losses, false positives, and false negatives. Below is a plot of the threshold values for each of the 50 points tested, and a grayscale representation of the threshold values (see Fig. 2). Additionally, total deviation and pattern deviation grids will be added to indicate if the point was less than 5%, 2%, 1%, or 0.5% of expected value once a normative database is established and validated. MD and PSD are listed for each eye. Fixation losses, false negatives, threshold values, and a grayscale map present raw data, whereas the rest of the reported information will be the result of analysis based on a normative database. The physician's identifying information appears at the bottom of the report.

### Subjects

In this pilot study, we present test data from 3 healthy volunteers with best corrected visual acuities of 20/25 or better in each eye with no known VF defects on SAP, and no known eye disease. This study was deemed exempt and approved as such by the institutional review board (IRB) at Vanderbilt University Medical Center. The study protocol adhered to the tenets of the Declaration of Helsinki. All patients were informed of the potential risks and benefits, and all signed an informed consent form before the protocol.

Participants were asked to remain seated for the duration of the test. They were asked to place the headset over their head and adjust the straps for the proper fit. Securing the goggles snugly was important so the images were crisp on the screen. The handpiece was placed in the participant's dominant hand and the participant was shown the “select” button. Each participant underwent testing with the T-24 protocol. Participants were instructed to select the “Click to Start” button on the screen and a brief tutorial was displayed. The patients were instructed to gaze at the red fixation target in the center of the screen and depress the “trigger” on the underside of the handpiece when a flash of light was seen. Participants were instructed to wear glasses as needed during the test.

Test results were stored along with basic identifying information and were accessed through a secure web portal. Data collected from each test included test duration per eye (mean test duration was calculated as total test time as a fraction of the number of eyes tested), total fixation losses, total false positives, and total false negatives. Mean threshold values were collected from each quadrant (Superior Temporal [ST], Superior Nasal [SN], Inferior Nasal [IN], and Inferior Temporal [IT]), and from the central 12 degrees (central), 12 to 24 degrees (pericentral), and of all the testing loci (global).

## Results

Six eyes of three subjects were tested (2 men and 1 woman; all were 30 years of age). One subject required and wore refractive correction during the test.

All participants completed the test successfully. Simultaneous testing of both eyes resulted in a mean test duration of 532.6 ± 13.5 (SD) seconds (8 minutes and 52 seconds) or roughly 266.3 ± 6.8 seconds (4 minutes 26 seconds) per eye.

Fixation losses were detected in 2 of 12 tests in the right eyes and 0 of 12 in the left. False positive responses occurred with frequency of zero in each eye of one participant, 0.97% in the right eye of the second participant and 3.45% in the left eye, and 2.3% in the right and left eyes of the third participant. There were no false negatives recorded.

**Figure 3.**
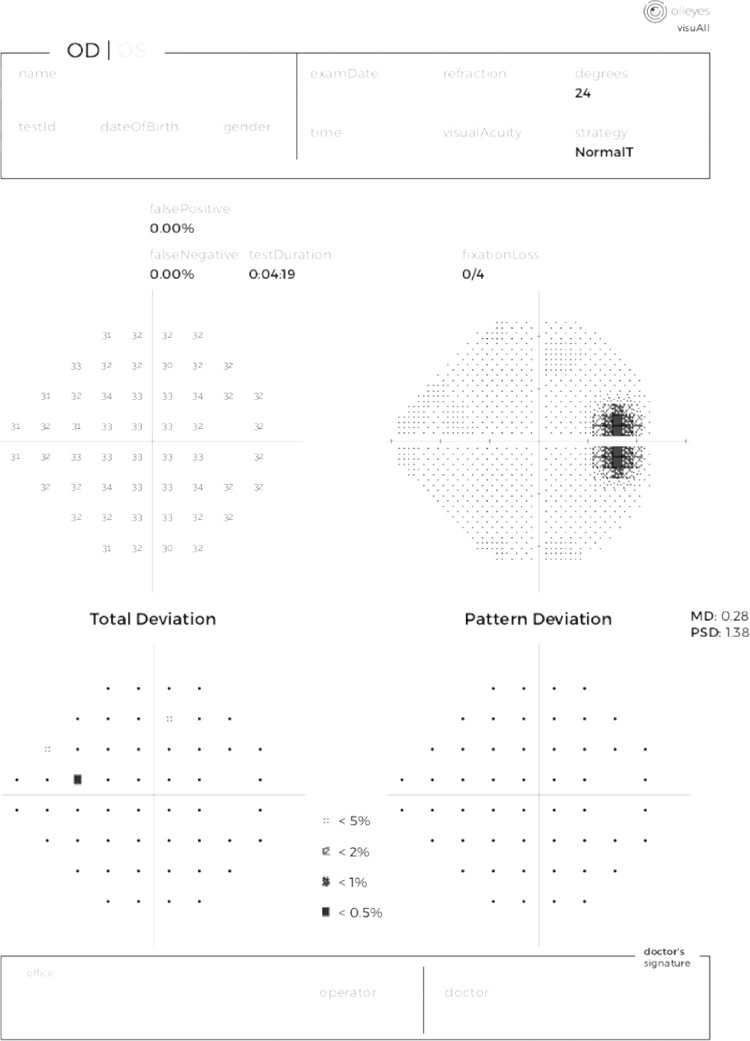
Report generated after completion of a test using the VisuALL.

Threshold values are elaborated in the Table and illustrated in Figure 3. The means of the threshold measurements at each sector were comparable. The loci tested within the central 12 degrees had the highest sensitivity (31.0 ± 0.6 dB), a finding expected in this healthy population.

**Table.** Mean Threshold Values (In dB of Attenuation) With Standard Deviation at Each Sector Tested

	Subject 1		Subject 2		Subject 3	
	Mean Sensitivity, dB (SD)		Mean Sensitivity, dB (SD)		Mean Sensitivity, dB (SD)	
Sector	OD	OS	OD	OS	OD	OS
Superior temporal	32.2 (0.9)	32.6 (0.8)	32.2 (0.9)	30.0 (2.5)	32.0 (0.9)	32.0 (0.4)
Superior nasal	32.2 (0.9)	31.4 (3)	32.0 (1.4)	32.1 (0.8)	32.0 (1)	31.6 (2.1)
Inferior nasal	32.5 (0.8)	32.2 (1.1)	32.0 (1.1)	31.6 (1.3)	32.4 (0.9)	31.8 (1.1)
Inferior temporal	32.3 (1)	32.8 (0.8)	32.0 (1)	29.5 (2)	31.9 (1)	32.0 (0.8)
Global	32.3 (0.9)	32.2 (1.8)	32.0 (1.1)	30.8 (2)	32.1 (0.9)	31.8 (1.3)
Central	33.2 (0.5)	33.3 (0.6)	33.0 (1)	31.8 (1.3)	32.8 (0.9)	32.6 (1)
Pericentral	31.9 (0.7)	31.7 (1.9)	31.6 (0.9)	30.4 (2.1)	31.8 (0.8)	31.4 (1.2)

## Discussion

The VisuALL is a portable automated perimeter that uses a virtual interface that has potential to provide an immersive testing experience for patients. The VisuALL has two displays (one for each eye) allowing it to test both eyes simultaneously but separately with similar test duration to other perimeters. Mean test times in healthy participants tested with the HFA's automated static SITA Faster, SITA Fast, and SITA Standard were 171.9, 247.0, and 369.5 seconds per eye, respectively.^10^ Meanwhile, those tested with the Octopus’ 3 degrees/second, 5 degrees/second, and 10 degrees/second were 267.6, 139.8, and 138 seconds per eye, respectively. In our study, participants had an estimated monocular mean test time of 266.3 seconds.

VR technology is becoming increasingly available. Approximately 7 million VR headsets have been sold since they were first introduced to the general public in 2010. Early applications revolved around entertainment and broadened to include medical education,^11^ flight training,^12^ driver training, rehabilitation for post-traumatic stress disorder (PTSD)^13^ and Alzheimer's disease,^14^ and surgical training.^15^ The increased presence of VR in ophthalmology could make it easier for healthcare providers to individualize management; VR tools can be adapted to a patient instead of requiring patients to conform to analog instruments.

This device's portability may contribute to increased efficiency in a typical ophthalmology practice; multiple test subjects can be assessed simultaneously using the same server in a single, well compartmentalized room, by a single technician. Doctor-patient interactions may also change from centralized to more individual settings, providing more options for providers to reach marginalized and underserved communities.

Telemedicine has been shown to be beneficial to patients for clinical and economic reasons. Many mature domains for remote monitoring in ophthalmology exist, but few options are available to patients with glaucoma,^16^^,^^17^ and the available options require patients to travel to a designated testing center.^16^ Home monitoring has been successful for other conditions,^18^ and previous studies have shown that patients with glaucoma are capable of performing self-tonometry using a rebound tonometer.^19^ Testing with the VisuALL does not require a specific testing location and, therefore, may fill gaps present in at-home glaucoma monitoring. It may enhance healthcare access and quality by extending the reach of eye care services leading to improved clinical outcomes. Many patients with glaucoma may benefit from increased VF testing; as tracking disease progression becomes easier,^20^ providers may be able to watch for progression more closely.

At-home glaucoma monitoring has other added benefits; more frequent testing may allow patients to overcome learning curves more quickly (which generally occurs after 3–4 fields).^7^ An Increase in comfort may shorten test duration; as an example, fewer fixation losses were noted as patients could move their head freely while wearing a similar type of VF headset.^21^ At-home testing impacts quality of life, especially for patients for whom transportation to appointments is challenging.

Limitations of this study include its small sample size, and single center enrollment. Further adequately powered studies that assess its validity and reliability are required before a claim on its clinical utility can be made.

The VisuALL is an immersive, VR-based, automated perimeter that has potential to address some of the limitations inherent to other popular perimetric devices. This report shows that healthy, perimetrically experienced participants can complete testing with this device with ease. Potential advantages of the VisuALL are its adaptability, portability, and efficiency for patients. This device may fill the gap present in at-home glaucoma monitoring and expand the reach of glaucoma management.
